# Celecoxib Up Regulates the Expression of Drug Efflux Transporter ABCG2 in Breast Cancer Cell Lines

**Published:** 2014

**Authors:** Fatemeh Kalalinia, Fatemeh Elahian, Fatemeh Mosaffa, Javad Behravan

**Affiliations:** a *Biotechnology Research Center, School of Pharmacy, Mashhad University of Medical Sciences, Mashhad, IRAN.*; b *Department of Pharmaceutical Biotechnology, School of Pharmacy, Mashhad University of Medical Sciences, Mashhad, IRAN.*; c *Department of Pharmaceutical Biotechnology, School of Pharmacy, Zanjan University of Medical Sciences, Zanjan, IRAN.*

**Keywords:** Multidrug resistance, ATP-binding cassette transporter, ABCG2, MCF-7, Celecoxib

## Abstract

Elevated expression of the drug efflux transporter ABCG2 seems to correlate with multidrug resistance of cancer cells. Specific COX-2 inhibitor celecoxib has been shown to enhance the sensitivity of cancer cells to anticancer drugs. To clarify whether ABCG2 inhibition is involved in the sensitizing effect of celecoxib, we investigated whether the expression of ABCG2 in breast cancer cell lines, could be modulated by celecoxib. The expression of the multidrug resistant gene (ABCG2) at mRNA and protein level was detected by real-time quantitative reverse transcription-polymerase chain reaction and flow cytometry analysis, respectively. Among three human breast cancer cell lines ABCG2 and COX-2 were highly expressed in MCF7-MX and MDA-MB-231 cells, respectively. The COX-2 inhibitor celecoxib up-regulated the expression of ABCG2 mRNA in MCF-7 and MCF7-MX cells, which was accompanied by increased ABCG2 protein expression. While celecoxib was able to block the 12-O-tetradecanoylphorbol-13-acetate (TPA)-mediated increase in COX-2 expression in MDA-MB-231 cells, it increased the expression of ABCG2 up to 4.27 times to the control level at mRNA level and with less intensity at protein level. Our findings provide evidence that celecoxib up-regulates ABCG2 expression in human breast cancer cells and proposed that ABCG2 is not involved in chemosensitizing effects of celecoxib.

## Introduction

Cyclooxygenases-2 (COX-2) plays an important role in the prostaglandins production involved in pathophysiological processes like inflammation and carcinogenesis (1, 2). COX-2 has been characterized as a critical prognostic factor in numerous solid tumors (3). Numerous studies showed that specific COX-2 inhibitors (coxibs) enhance the efficacy of different anticancer therapy methods through inhibition of cell cycle progression, induction of apoptosis, inhibition of angiogenesis and decreased invasive potential of tumor cells (4-6). Another mechanism by which COX-2 inhibitors could sensitize cells to chemotherapeutic drugs is functional blockade of membrane transporter proteins of the ATP binding cassette transporter (ABC-transporter) family (7, 8).

The development of intrinsic or acquired resistance to a wide variety of anticancer drugs is a major obstacle to successful cancer chemotherapy. One of the mechanisms by which human cancers develop multidrug resistance is the overexpression of efflux transport proteins on the plasma membrane of cancer cells. ATP-binding cassette sub-family G member 2 (ABCG2), a novel known protein, is an efflux pump which transports a variety of xenobiotics and endogenous compounds across cellular membranes. Tissue localization of ABCG2 indicates that ABCG2 plays an important role in absorption, distribution and elimination of its substrates (9, 10). Alteration in ABCG2 expression and activity can significantly affect the disposition of the transporter drug substrates, so it's over expression in cancer cells is responsible for decreasing in drug concentration within the cell and a reduced cancer-chemotherapy efficacy (9, 11). 

Previously, we showed that proinflammatory cytokines (IL-1β and TNF-α) significantly increased the levels of ABCG2 mRNA, protein expression and function, while dexamethasone (an anti-inflammatory drug) reduced its expression and activity (12-16). The present study aimed to examine the relationship between the inhibition of COX-2 and expression of ABCG2 in parental and resistant breast cancer cell lines. Understanding the relationship between COX-2 and ABCG2 may open novel perspectives in cancer therapy, in which, coxibs could be employed as a chemotherapy-supporting treatment. Introducing coxibs into the treatment regimen will then augment efficiency of chemotherapy and maybe helpful in prevention or inhibition of the development of the multidrug resistance (MDR) phenomenon.

## Experimental

*Chemicals and antibodies*

 TPA (12-O-tetradecanoylphorbol-13-acetate), BSA and penicillin–streptomycin were purchased from Sigma–Aldrich (Germany). Celecoxib was generousely provided by Daru Pakhsh (Tehran, Iran). RPMI 1640 with L-glutamine and FBS were purchased from Biosera (UK) and Gibco (USA), respectively.

*Cell culture*

The ABCG2-overexpressing, mitoxantrone-resistance epithelial breast cancer cell line, MCF-7/MX, and its parental line, MCF-7 were generously provided by Dr. Erasmus Schneider (Wadsworth Center, New York State Department of Health, USA) and MDA-MB-231 (COX-2 expressing cell line) was generously provided by Dr. Mohammad Kazem Koohi (Faculty of Veterinary Medicine, university of Tehran, Iran). Cells were cultured in RPMI-1640, supplemented with 2 mM of L-glutamine, heat inactivated FBS 10% (v/v), penicillin (50 U/mL), and streptomycin (50 μg/mL) at 37 °C in humidified air containing CO_2 _5%. Since FBS has been shown to induce COX-2 (due to the presence of growth factors (17)), before treatment, subconfluent cells were washed with PBS and changed to serum-free medium containing BSA 0.1% (w/v) for 24 h. **Mitoxantrone** (MX) (100 nM) was added to MCF-7/MX cells to maintain the multidrug-resistant phenotype. MCF-7/MX cells were cultured on MX free for at least 7 days prior to experiments.

*Reverse transcription-real-time polymerase chain reaction (RT-PCR)*

Total cellular RNA was extracted using a High Pure RNA Isolation Kit from Roche Applied Science (Cat. No. 11828665001). The eluted RNA samples were stored at –80 °C for later analysis or directly used in RT-PCR. In order to evaluate the effects of celecoxib on ABCG2 and COX-2 expression, comparative Ct methods using EXPRESS One-Step SYBR® GreenER™ Universal (Invitrogen) were performed and analyzed on a Real time cycler Mx3000P™ Stratagen (Stratagen, USA ). The primers had the following sequences: ABCG2: 5´- TATCAATGGGATCATGAAACCTGG-3´ (forward) and 5´-GCGGTGCTCCATTTATCAGAAC-3´(reverse); COX-2, 5´-AATCATTCACCAGGCAAATTG- 3´ (forward ) and 5´-TCTGTACTGCGGGTGGAACA- 3´ (reverse ); β-actin: 5´-TCATGAAGTGTGACGTGGACATC-3´ (forward) and 5´-CAGGAGGAGCAATGATCTTGATCT-3´ (reverse). Relative mRNA levels of the target gene in each sample were normalized to its β-actin content. The results were expressed as ratio of relative quantity to calibrator of the treated samples divided by relative quantity to calibrator of untreated sample.

*Flow cytometric detection of COX-2 protein expression*

For intracellular detection of COX-2 by flow cytometry, breast cancer cells were fixed and permeabilised using the Leucoperm™ fixation and permeabilization solution, following the manufacturer's instructions (18). Thereafter, the cells were blocked with BSA 10 % (w/v) and incubated with 50 ng of Phycoerythrin (PE) -conjugated anti-human COX-2 for 30 min. Finally samples were washed, resuspended in PBS and analysed by PartecTM cytometer with 585/42 band pass filter (FL2). Relative expression of COX-2 was calculated, comparing the values of mean fluorescence intensity (MFI) of treated samples to the values of MFI of untreated sample.

*Flow cytometric detection of ABCG2 protein expression*

The ABCG2 protein expression was measured with the BXP-21 monoclonal antibodies, which recognize an internal epitope of the ABCG2 protein as we previously described (12, 14). In brief, after fixation and permeabilization, the cells were blocked with bovine serum albumin (BSA) 10% (w/v) for 1 h at room temperature. Thereafter, the cells were incubated for 60 min on ice with anti-ABCG2 mAb BXP-21 (1:100). Cells were washed with phosphate buffered saline/bovine serum albumin and incubated on ice for 20 minutes with Fluorescein isothiocyanate (FITC) -conjugated goat anti-mouse antibody (1:50) to detect the primary anti-ABCG2 mAb. FITC fluorescence was measured on a Partec^TM^ cytometer with 530/30 nm band pass filter (FL1). Flow cytometry data were processed and analyzed using FloMax version 2.52 and WinMdi version 2.8 software. All assays were performed in at least three independent experiments. Relative expression of ABCG2 was determined as the ratio of MFI of treated samples divided by MFI of untreated sample.

*Statistical analyses*

Statistical analyses were carried out using SPSS 16.00 software. Results (mean ± S.D.) were reported as three independent experiments each performed in triplicate. The statistical significance of the results was calculated using multivariate analysis of variance (MANOVA) with Tukey’s post-hoc or Student’s t-test and p-values < 0.05 were considered significant.

## Results

*ABCG2 and COX-2 expression in MCF7 cells*

**Figure 1:**
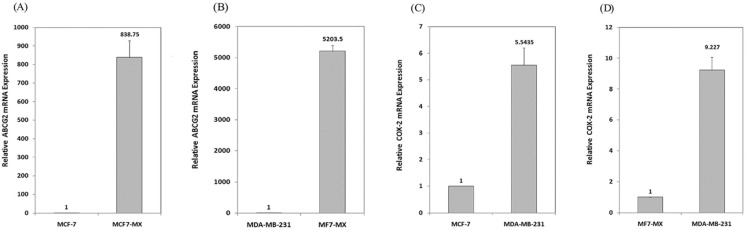
Basal expressions of ABCG2 and COX-2 mRNA in breast cancer cell lines were studied by real time RT-PCR. ABCG2 mRNA level is compared between drug resistant cell line MCF7-MX with MCF-7 (A) and MDA-MB-231 (B) cells. In addition, COX-2 mRNA level is compared between COX-2 overexpressing cell line MDA-MB-231 with MCF- 7 (C) and MCF7-MX (D). Real-time RT-PCR analysis was performed on total RNA extracted from cells. Values were normalized to the β-actin content of samples and expressed as mean ±SD (n = 3).

In order to assess ABCG2 and COX-2 expression in MCF7 cells, the expression of these two genes was evaluated in MCF7 and control cell lines (MCF7-MX and MDA-MB-231cells) by real time RT-PCR. As shown in Figure 1, ABCG2 mRNA level in resistant cell line MCF7-MX was ≈ 850 and 5200 times more than the ABCG2 mRNA level in MCF-7 and MDA-MB-231 cells, respectively. The expression of COX-2 in MDA-MB-231 cells was ≈ 5.5 and 9.2 times more than MCF-7 and MCF7-MX cells, respectively (Figure 1). These data are further strengthened by the results of flowcytometry analyses for ABCG2 and COX-2 protein expression (data not shown).

*Effects of celecoxib on COX-2 expression*

**Figure 2:**
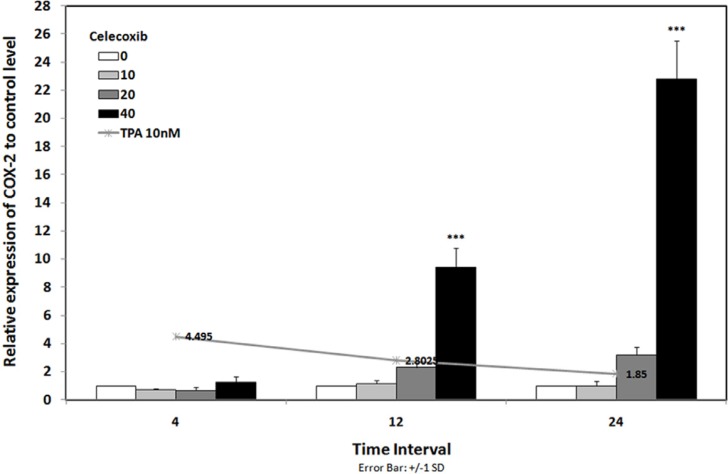
COX-2 mRNA expression in MDA-MB-231 cell line under treatment with and without celecoxib. Line chart shows the results of cells that were treated with TPA 10 nM lonely. Bar chart indicated the results of cells that were treated with TPA 10 nM in combination with celecoxib 0-40 µM for 4-24 h. Real-time RT-PCR analysis was performed on total RNA extracted from control and treated cells. Values were normalized to the β-actin content of samples. The results were expressed as the target/reference ratio of the treated samples divided by the target/reference ratio of the untreated control sample and expressed as mean ± SD (n = 3); *, p < 0.05; **, p < 0.01; ***, p < 0.001

**Figure 3:**
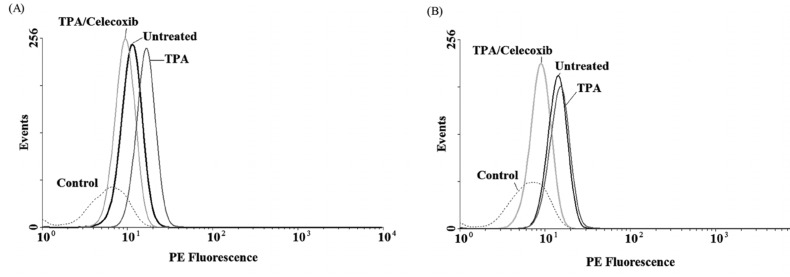
COX-2 protein expression in MDA-MB-231 cell lines under treatment with TPA, and celecoxib for 4 h (A) and 24 h (B). Cells were treated with or without TPA 10 nM, and celecoxib 40 µM and COX-2 protein expression were measured by flow cytometric assay, which developed for detection of COX-2. Briefly, after fixation and permeabilization, cells were blocked with BSA 10% (w/v) and incubated with PE- conjugated anti-COX-2 mAbs. COX-2 protein level expressed as mean fluorescence intensity (MFI) of PE-fluorescence of 10,000 cells that was quantified in histogram plots. Each histogram shows the overlay of the TPA treated sample (dark gray), TPA and celecoxib treated sample (ligh gray), untreated sample (black) and unstained control sample, which has been used to detect autofluorescence (broken light gray).

To evaluate the expression of COX-2 mRNA under celecoxib treatment, three cell lines were treated with non-toxic concentration of TPA (10 nM) and celecoxib (0-40 µM) for 4-24 h (15). Expression of COX-2 was studied using Real-time RT-PCR. We have previously reported that TPA was a potent stimulator of COX-2 expression in breast cancer cell lines; as it stimulates COX-2 expression up to 4.5 fold vs. control by 4 h in MDA-MB-231 cells (15). Figure 2 indicates that celecoxib reverses the effects of TPA on COX-2 expression to the control level in MDA-MB-231 by 4 h. Celecoxib significantly inhibited COX-2 expression by 12 and 24 h in MCF-7 and by 24 h in MCF7-MX (data not shown). To verify whether the inhibitory effects of celecoxib on COX-2 expression could be observed also in protein level, cells were treated for 4 and 24 h without or with TPA 10 nM, celecoxib 40 µM, or both. Then, the cells were incubated with the specific COX-2 antibody. As shown in Figure 3, MDA-MB-231 cells treated with TPA displayed an increased COX-2 protein expression, which was significantly reduced by co-treatment with celecoxib.

*Effects of celecoxib on ABCG2 gene expression*

**Figure 4:**
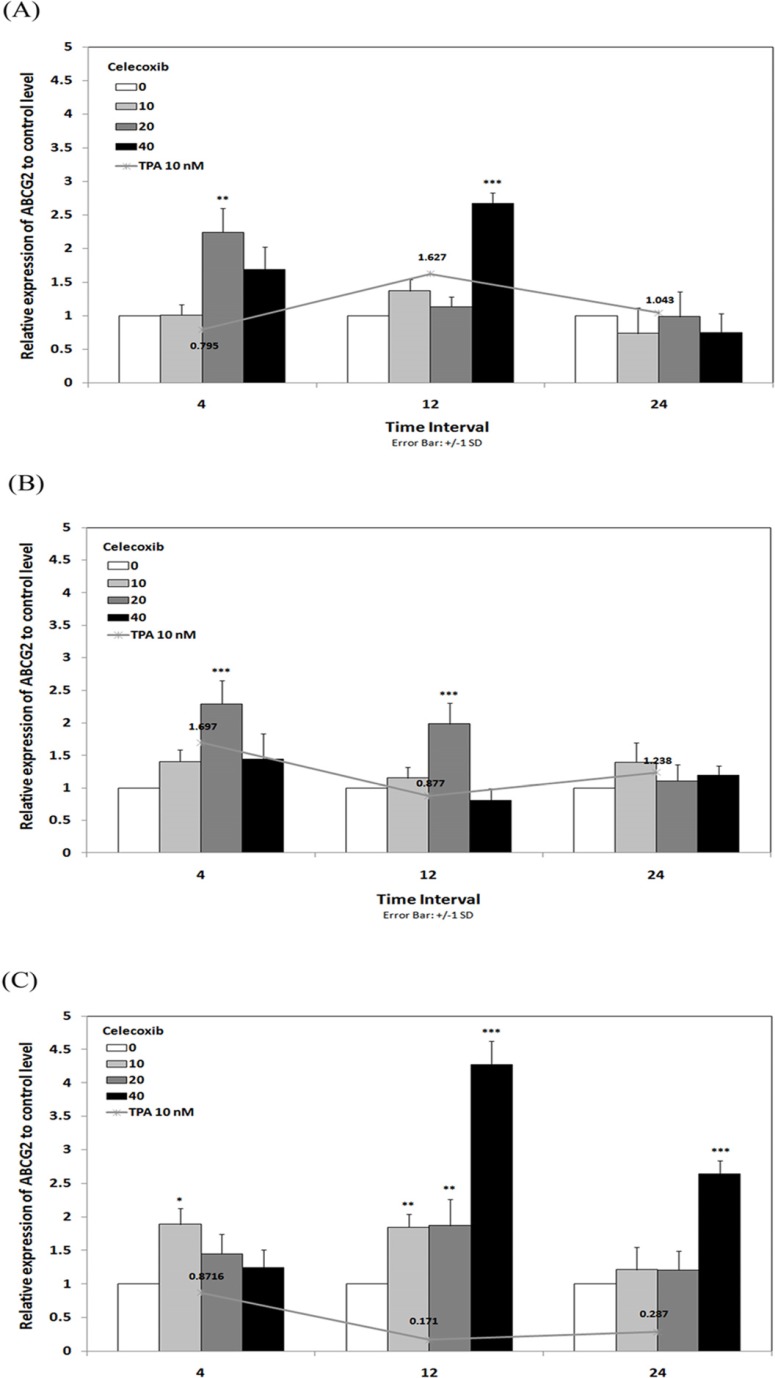
Effects of celecoxib on the levels of ABCG2 mRNA in MCF-7 (A), MCF7-MX (B), and MDA-MB-231 (C) cell lines. Cells were treated for 4, 12, and 24 h with TPA 10 nM alone (line chart) or in combination with celecoxib 0-40 µM (bar chart) and ABCG2 mRNA expression were measured by real-time RT-PCR using total RNA extracted from control and treated cells. Values were normalized to the β-actin content of samples. The results were expressed as the target/reference ratio of the treated samples divided by the target/reference ratio of the untreated control sample and expressed as mean ± SD (n = 3); *, p < 0.05; **, p < 0.01; ***, p < 0.001.

To verify whether ABCG2 gene expression could be influenced by incubation with celecoxib, real time RT-PCR analysis was performed on RNA from breast cancer cells previously treated for 4, 12 and 24 h with TPA 10 nM in combination with celecoxib 0-40 µM. The results showed that celecoxib enhanced the effects of TPA on ABCG2 expression in MCF-7 and MCF7-MX. In MCF-7 (Figure 4a), ABCG2 expression increased up to 1.62 times under treatment with TPA 10 nM for 12 h while combination treatment with celecoxib 40 µM / TPA 10 nM caused up to 2.67 times increased in ABCG2 expression to control level in the same incubation time. As shown in Figure 4b, celecoxib 20 µM induced ABCG2 expression from 1.69 times (under TPA 10 nM treatment) to 2.29 times to control level by 4 h in MCF7-MX cells. On the other hand, celecoxib reversed the TPA effects on ABCG2 expression in MDA-MB-231 cell line. We previously reported that TPA significantly reduced ABCG2 expression to about 0.2 times to control level by 12 and 24 h (18), while as shown in Figure 4c, celecoxib stimulated the expression of ABCG2 up to 4.27 times to control level by 12 h in MDA-MB-231 cells. 

*Influence of celecoxib on ABCG2 protein expression*

**Figure 5:**
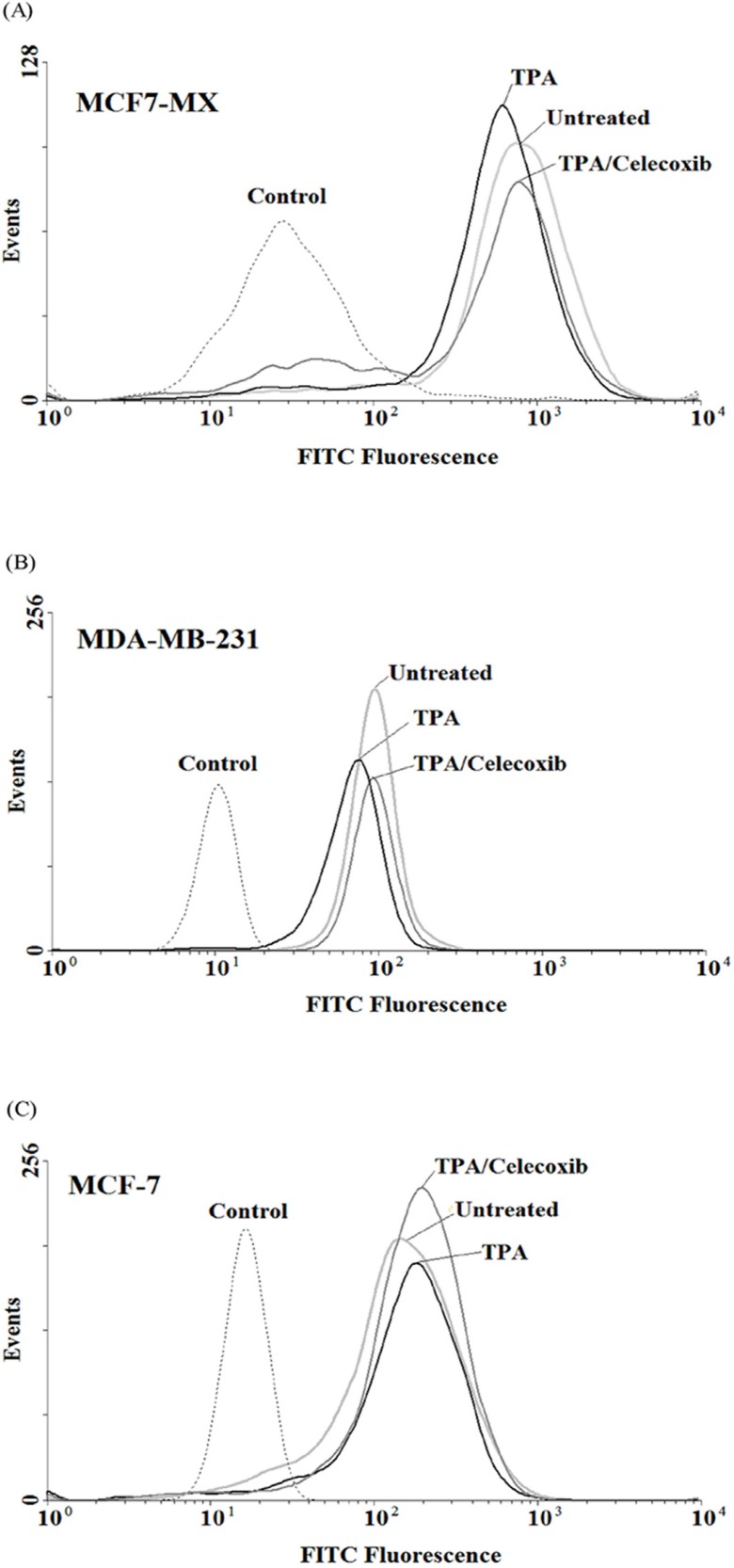
Effects of celecoxib on the expression of ABCG2 at protein levels in MCF7-MX (A), MDA-MB-231 (B), and MCF-7 (C) cell lines were studied by flow cytometry. Cells were fixed and permeabilized by formaldehyde and methanol, blocked with BSA and then incubated with primary monoclonal antibody BXP-21. After washing, Cells were incubated with a FITC-conjugated goat anti-mouse antibody. Fluorescence of 10,000 cells was quantified from histogram plots using the mean fluorescence intensity (MFI). Each histogram shows the overlay of the TPA treated sample (black), TPA and celecoxib treated sample (dark gray), untreated sample (ligh gray) and secondary antibody as negative control (broken light gray).

In order to verify whether the observed effects at mRNA level were mirrored by parallel changes in ABCG2 protein levels, flow cyotmtry analysis for ABCG2 was performed using breast cancer cells treated for 4 and 24 h with TPA 10 nM and celecoxib 40 µM. We previously showed that TPA could cause a small increase in ABCG2 expression in MCF-7, on the contrary, treatment of MCF7-MX and MDA-MB-231 cells with TPA resulted in a small decrease in ABCG2 protein expression (18). As indicated in Figure 5, celecoxib reversed the inhibitory effects of TPA on ABCG2 protein expression and increased its expression to the basal level in MCF7-MX and MDA-MB-231. Co-treatment of MCF-7 cells with TPA and Celecoxib caused increased ABCG2 protein expression to a small amount more than TPA lonely (Figure 5c).

## Discussion

ABCG2 is an important member of the ABC- transporter family, which functions as pumps to extrude anticancer drugs from cancer cells, by this means causing MDR in cancer patients (9). This study tested the hypothesis that COX-2 inhibitor celecoxib plays a role in regulating ABCG2 expression in human breast cancer cells. Recent studies indicated that the activation of the cyclooxygenase system is correlated with the increased expression of MDR1 in acute myeloid leukemia HL-60 cells, primary rat hepatocytes, renal rat mesangial cells as well as breast cancer patients (7, 19-21). While, selective COX-2 inhibitors have been shown to increase sensitivity of cancer cells to chemotherapy via reduction of drug resistance in human patients with AML, breast, ovarian and colon cancer (3, 19, 22, 23). On the other hand, in this study we showed that celecoxib up-regulated ABCG2 mRNA expression in drug sensitive MCF-7 cells. 

Our finding indicated that celecoxib reduced TPA-induced COX-2 mRNA expression at 10 µM while treatment with celecoxib 40 µM resulted in a significant overstimulation of COX-2 expression. On the other hand, celecoxib 40 µM inhibits TPA induced COX-2 expression at protein level (Figure 2 and 3). Our results are in good agreement with Niederberger *et al*. who showed that celecoxib 1 µM slightly reduced the IL-1β-induced COX-2 expression at both mRNA and protein level but increased COX-2 mRNA and protein expression at 50 µM. It has been shown that transcription factor NF-κB regulates the transcription of the COX-2 gene. On the other hand, high concentrations of celecoxib induced the activation of NF-kB, so activated NF-kB would stimulate the transcription of NF-kB-dependent genes such as COX-2 (24).

In this study, we reported that treatment of MCF-7 ,MCF7-MX and MDA-MB-231 cells with specific COX-2 inhibitor celecoxib up-regulates ABCG2 expression at both mRNA and protein levels. In the same way, Zrieki et al. showed that treatment of human colorectal Caco-2 cell line with COX-1/ COX-2 inhibitor naproxen led to an stimulation of ABCG2 expression corresponding to the significant decrease of rodamine 123 (Rho123) retention achieved in activity study. In contrast, treatment with selective COX-2 inhibitors nimesulide did not influence the expression of ABCG2 in protein level (25). Another study indicated that sulindac induced MRP1 and MRP3 gene expression via a ROS-related, COX-2-independent mechanism in human colorectal cancer cell line HCT15 (26). Kang et al. reported that enforced COX-2 expression in human lung carcinoma cell line H460 did not cause increase in MRP1 expression, while the celecoxib COX-2 inhibitor reduced the expression and function of MRP1 protein. They suggested that celecoxib down-regulated MRP1 expression with a COX-independent mechansim (27). In contrast, several studies have shown that specific COX-2 inhibitors could prevent or reduce the development of chemoresistance phenotype by downregulation of the expression and activity of P-glycoprotein (MDR1) (8, 26, 28-30). These inconsistencies may be due to the diversity among species, tissue origins, or cell types. 

In conclusion, our findings provide evidence that selective COX-2 inhibitor celecoxib induces ABCG2 mRNA and protein expression in the sensitive and drug resistant MCF7 breast cancer cell lines. These data may indicate a possible COX-2 independent mechanism for the stimulatory effect of celecoxib on ABCG2. Further studies are needed to determine whether other COX-2 selective inhibitors will show the similar effects to celecoxib and to clarify the molecular mechanism by which they induce ABCG2 expression.
